# Association Between Maternal Exposure to Lead, Maternal Folate Status, and Intergenerational Risk of Childhood Overweight and Obesity

**DOI:** 10.1001/jamanetworkopen.2019.12343

**Published:** 2019-10-02

**Authors:** Guoying Wang, Jessica DiBari, Eric Bind, Andrew M. Steffens, Jhindan Mukherjee, Romuladus E. Azuine, Gopal K. Singh, Xiumei Hong, Yuelong Ji, Hongkai Ji, Colleen Pearson, Barry S. Zuckerman, Tina L. Cheng, Xiaobin Wang

**Affiliations:** 1 Center on the Early Life Origins of Disease, Department of Population, Family, and Reproductive Health, Johns Hopkins Bloomberg School of Public Health, Baltimore, Maryland; 2 Division of Research, Office of Epidemiology and Research, Maternal and Child Health Bureau, Health Resources and Services Administration, Rockville, Maryland; 3 Metals Laboratory, Environmental and Chemical Laboratory Services, State of New Jersey Department of Health, Trenton; 4 Office of Health Equity, Health Resources and Services Administration, Rockville, Maryland; 5 Department of Biostatistics, Johns Hopkins Bloomberg School of Public Health, Baltimore, Maryland; 6 Department of Pediatrics, Boston University School of Medicine and Boston Medical Center, Boston, Massachusetts; 7 Division of General Pediatrics and Adolescent Medicine, Department of Pediatrics, The Johns Hopkins University School of Medicine, Baltimore, Maryland

## Abstract

**Importance:**

The first pediatric lead screening typically occurs at 1-year well-child care visits. However, data on the extent of maternal lead exposure and its long-term consequences for child health are lacking.

**Objective:**

To investigate the associations between maternal red blood cell (RBC) lead levels and intergenerational risk of overweight or obesity (OWO) and whether adequate maternal folate status is associated with a reduction in OWO risk.

**Design, Setting, and Participants:**

Prospective birth cohort study. The analysis was conducted from July 14, 2018, to August 2, 2019, at Johns Hopkins Bloomberg School of Public Health. This study included 1442 mother-child pairs recruited at birth from October 27, 2002, to October 10, 2013, and followed up prospectively at Boston Medical Center.

**Main Outcomes and Measures:**

Child body mass index (BMI) *z* score, calculated according to US national reference data, and OWO, defined as BMI at or exceeding the 85th percentile for age and sex. Maternal RBC lead levels and plasma folate levels were measured in samples obtained 24 to 72 hours after delivery; child whole-blood lead level was obtained from the first pediatric lead screening.

**Results:**

The mean (SD) age of mothers and children was 28.6 (6.5) years and 8.1 (3.1) years, respectively; 50.1% of children were boys. The median maternal RBC lead level and plasma folate level were 2.5 (interquartile range [IQR], 1.7-3.8) μg/dL and 32.2 (IQR, 22.1-44.4) nmol/L, respectively. The median child whole-blood lead level and child BMI *z* score were 1.4 (IQR, 1.4-2.0) μg/dL and 0.78 (IQR, −0.08 to 1.71), respectively. Maternal RBC lead level was associated with child OWO risk in a dose-response fashion, with an odds ratio (OR) of 1.65 (95% CI, 1.18-2.32) for high maternal RBC lead level (≥5.0 μg/dL) compared with low maternal RBC lead level (<2.0 μg/dL). Child OWO was highest among children of OWO mothers with high RBC lead levels (adjusted OR, 4.24; 95% CI, 2.64-6.82) compared with children of non-OWO mothers with low RBC lead levels. Children of OWO mothers with high RBC lead levels had 41% lower OWO risk (OR, 0.59; 95% CI, 0.36-0.95; *P* = .03) if their mothers had adequate plasma folate levels (≥20.4 nmol/L) compared with their counterparts.

**Conclusions and Relevance:**

In this sample of a US urban population, findings suggest that maternal elevated lead exposure was associated with increased risk of intergenerational OWO independent of postnatal blood lead levels. Adequate maternal folate status appeared to be associated with lower OWO risk. If confirmed by additional studies, these findings have implications for prenatal lead screening and management to minimize adverse health consequences on children.

## Key Points

**Question:**

Is maternal lead exposure associated with intergenerational risk of overweight or obesity and is folate associated with a reduction in these risks?

**Findings:**

In a cohort study of 1442 mother-child pairs, lead was detectable in all maternal samples. Children whose mothers had elevated red blood cell lead levels (≥5.0 μg/dL) were more likely to be overweight or obese.

**Meaning:**

In this sample of a US urban population, maternal lead exposure was widespread and associated with higher intergenerational risk of overweight or obesity, but adequate maternal folate status appeared to mitigate such risks.

## Teaser

This cohort study investigates the associations between maternal red blood cell lead levels and intergenerational risk of overweight or obesity and whether adequate maternal folate status is associated with a reduction in this risk.

## Introduction

Despite concerted efforts to decrease maternal and child obesity rates, obesity prevalence in the United States remains high, and low-income urban minority populations are disproportionately represented.^1^ The results of experimental studies suggest that the obesity epidemic could in part be due to chemical exposures during sensitive and vulnerable windows of development, mainly in utero and infancy.^2^ The role of the maternal environment in shaping metabolic processes and disease risk later in life has been widely reported.^3,4,5,6^ Fetal programming during gestation results in irreversible changes to the body structure, function, and metabolism of the fetus, which may lead to a vicious cycle of intergenerational amplification of obesity.^7,8^ One may postulate that the consequences of maternal obesity accumulate over successive generations to shift the population distribution of weight toward the heavy side, but little is known about what factors can enhance or mitigate the intergenerational link.

Lead is a highly toxic metal that previously had been widely used in numerous consumer goods, leading to widespread contamination of air, water, and soil. Because of its fetal toxic effects and multiorgan detrimental associations across a wide range of exposures without a clear threshold, lead is among the top 10 chemicals of major global public health concern.^9^ Although blood lead levels for the average population have decreased significantly with improvements in environmental policies,^10^ exposure to lead is still ubiquitous in the United States^11^ and remains a social ecodisadvantage.^12^ More alarming is that bone lead, which accounts for 90% of the total body burden, is mobilized during pregnancy and lactation, resulting in a source of in utero lead exposure.^13^ Because bone lead stores persist for decades,^14^ women and their infants may be at risk for continued exposure long after exposure to external environmental sources.

It is well recognized that maternal exposure to lead impairs both maternal health and infant neurodevelopmental outcomes.^15^ Animal models suggest that higher perinatal blood lead levels are associated with increases in the body weight of offspring^16^; however, the long-term consequences of maternal lead exposure on human child overweight or obesity (OWO) risk are inconclusive.^17,18^ To date, there is a lack of prospective birth cohort studies to delineate dose-response associations between prenatal lead exposure and OWO risk in childhood with or without maternal OWO. Emerging evidence suggests that adequate maternal folate status is beneficial to child metabolic health and biomarkers of adiposity^19^ and can have protective consequences for the intergenerational risk of obesity.^20,21^ However, it is unknown if adequate maternal folate status confers protection in the setting of maternal lead exposure.

The objectives of this study were to examine whether maternal lead exposure is associated with intergenerational OWO risk and whether adequate maternal folate status is associated with reduced risk in such a setting. This study represents a convergence of multidisciplinary inquiry to better understand risk and protective factors of the intergenerational link of OWO.

## Methods

This study followed the Strengthening the Reporting of Observational Studies in Epidemiology (STROBE) reporting guideline. The study protocol was approved by the institutional review boards of Boston Medical Center and Johns Hopkins Bloomberg School of Public Health.

### Study Population

This prospective birth cohort study included 1442 mother-child pairs who were recruited at birth from October 27, 2002, to October 10, 2013, and followed up prospectively thereafter at Boston Medical Center. The study sample is a subset of the Boston Birth Cohort. Detailed information on participant enrollment has been described previously.^22^ Briefly, the mother-infant pairs were enrolled 24 to 72 hours after delivery. After obtaining written informed consent, mothers were interviewed using a standardized questionnaire by trained research staff. Pertinent clinical information was obtained by a review of maternal and infant medical records, including prenatal ultrasonographic reports, laboratory reports, pregnancy complications, labor and delivery course, and birth outcomes. Of a total of 3163 children followed up in the Boston Birth Cohort, 1551 mothers had lead levels in red blood cells (RBCs) measured. Overall, 109 children were excluded because of the lack of body mass index (BMI) data between ages 2 and 15 years. The 1442 children included in the study were those who completed at least 1 well-child care visit after age 2 years and whose mothers had measured RBC lead levels (eFigure 1 in the Supplement). The included and excluded groups were comparable except for race/ethnicity and smoking status (eTable 1 in the Supplement).

### Ascertainment of Maternal RBC Lead Levels and Child Whole-Blood Lead Levels

Maternal lead levels in RBC samples obtained 24 to 72 hours after delivery were measured using inductively coupled plasma mass spectrometry (8900 ICP-QQQ; Agilent Technologies Inc) by a Centers for Disease Control and Prevention–certified laboratory for the National Biomonitoring Program (Metals Laboratory, Environmental and Chemical Laboratory Services, State of New Jersey Department of Health, Trenton) according to standard protocols for quality control and assurance. An additional 89 duplicate blinded samples were interspersed, and the coefficient of variation (CV) was less than 5.0%. Child whole-blood lead levels were obtained from medical records of the first pediatric lead screening, which typically occurs at 1-year well-child care visits.

### Ascertainment of Maternal Plasma Folate Levels and Child Metabolic Biomarkers

Maternal plasma folate levels were measured in archived plasma samples obtained 24 to 72 hours after delivery by a commercial laboratory via chemiluminescent immunoassay (MAGLUMI 2000; Snibe Co, Ltd), with an interassay CV of less than 4%.^23^ We defined adequate maternal folate status as plasma folate levels of at least 20.4 nmol/L (cut point previously defined^19^). Levels of child plasma insulin (a marker of insulin resistance^22^) and leptin (a marker of adiposity^24^) during early childhood were determined using sandwich immunoassays based on flowmetric technology (Luminex 200; Luminex Corp), with an interassay CV of 4.0% and 4.5%, respectively.^19,22^

### Definition of Maternal Characteristics

Maternal epidemiological variables, including age at time of delivery, educational level, race/ethnicity, smoking status, parity, and prepregnancy weight and height, were based on answers to the maternal questionnaire interview. Maternal race/ethnicity was classified as black (including African American or Haitian), Hispanic, or other (including white, Asian or Pacific Islander, >1 race, or other). Maternal pregnancy complications, including diabetes (gestational diabetes or preexisting diabetes) and hypertensive disorders (preeclampsia, eclampsia, or chronic hypertension, and the HELLP syndrome [hemolysis, elevated liver enzymes, and low platelets)), were obtained through a standardized review of medical records. Maternal prepregnancy BMI was calculated as prepregnancy weight in kilograms divided by height in meters squared and further dichotomized as non-OWO (BMI <25) and OWO (BMI ≥25). Gestational age was estimated based on both the first day of the last menstrual period, as recorded in the maternal medical record, and early (<20 weeks) prenatal ultrasonographic results, as detailed in a previous publication.^22^

### Assessment of Child’s Birth Outcomes and Breastfeeding Status

We abstracted the clinical measurement of birth weight from the medical records. We categorized fetal growth into the following 3 groups: small for gestational age (gestational age–specific birth weight <10th percentile), large for gestational age (birth weight >90th percentile), and appropriate for gestational age (birth weight in the 10th-90th percentiles for gestational age) according to an established local reference population and taking into account infant gestational age, sex, and race/ethnicity.^22^ Information regarding infant breastfeeding history was primarily assessed within the first 2 years of follow-up visits and grouped into formula exclusively, both formula and breastfed, or breastfed exclusively.^19^

### Definitions of the Primary Outcomes of Child BMI and OWO in Childhood

Child weight and height were measured by trained medical staff during well-child care visits as documented in the medical records. Careful data cleaning of weight and height data was performed. We first removed extreme or biologically implausible values. Then, outliers or erroneous weight and height values were identified based on growth curves. When possible, we corrected erroneous weight and height values; otherwise, the points were deleted. Child BMI *z* scores and percentiles were calculated using US national reference data for age and sex.^25^ Overweight or obesity was defined as BMI at or exceeding the 85th percentile for age and sex.^26^ The Boston Birth Cohort uses rolling enrollment, so the length of postnatal follow-up and number of well-child care visits varied greatly. It is known that OWO at older age is more likely to persist into adulthood. Therefore, we chose the last well-child care visit child BMI *z* scores and OWO as the end points of this report.

### Statistical Analysis

The analysis was conducted from July 14, 2018, to August 2, 2019, at Johns Hopkins Bloomberg School of Public Health. Unadjusted *P* values for trend across maternal RBC lead level quartiles were calculated by the Mantel-Haenszel χ^2^ test for categorical variables and by linear regression for continuous variables. The associations between maternal RBC lead levels and child outcomes (child BMI *z* scores and OWO) were summarized using locally weighted regression smoothing plots (implemented by PROC LOESS in SAS).

We used multivariable linear or logistic regression models to assess whether there was an independent association between maternal RBC lead levels or child whole-blood lead levels and child BMI *z* scores and OWO after adjustment for important covariates, including maternal educational level, race/ethnicity, smoking status, parity, diabetes, hypertensive disorder, fetal growth, and breastfeeding status. Twenty-seven of 1442 children (1.9%) were missing data for breastfeeding status, for which multiple imputation methods were used to replace the missing information. With a sample size of 1442, we had greater than 80% power to detect the associations for both continuous and binary outcomes.

In addition, we evaluated the joint risk attributed to maternal prepregnancy OWO and the lead levels. We tested the interaction between maternal prepregnancy OWO (as a binary variable) and RBC lead levels (as a continuous variable) on child BMI *z* score and odds of OWO by adding a multiplicative term in the models. Association modifications were assessed with the likelihood ratio test using an a priori α level of .05. We also investigated whether the association between maternal RBC lead level and child OWO risk differs by maternal plasma folate level.

To explore the potential biological pathways, we performed mediation analysis using the %MEDIATE macro.^27^ The mediation proportion quantified the extent to which the association between maternal exposure to lead and child OWO risk is mediated through lead exposure relative to the child’s plasma insulin and leptin levels in early childhood.

Finally, to examine the robustness of the results and biological plausibility, we conducted a series of sensitivity analyses. These included the following subgroup analyses: analyses stratified by child age at outcome assessments (eg, 2-5 years, 6-9 years, and 10-15 years), black children only, term births only, sequential models adding more covariates of interest, and propensity score–matched sensitivity analyses^28^ to address potential unmeasured confounders. *P* < .05 was regarded as statistically significant. All *P* values were from 2-sided tests, and analyses were performed using statistical software (SAS, version 9.4; SAS Institute Inc).

## Results

Of the 1442 study children, 722 (50.1%) were boys, mothers of 967 children (67.1%) were black, and mothers of 291 children (20.2%) were Hispanic. The mean (SD) age of mothers and children was 28.6 (6.5) years and 8.1 (3.1) years, respectively. The median maternal RBC lead level and plasma folate level were 2.5 (interquartile range [IQR], 1.7-3.8) μg/dL (to convert lead level to micromoles per liter, multiply by 0.0483) and 32.2 (IQR, 22.1-44.4) nmol/L, respectively. The median child whole-blood lead level and child BMI *z* score was 1.4 (IQR, 1.4-2.0) μg/dL and 0.78 (IQR, −0.08 to 1.71), respectively. The distribution of maternal RBC lead levels in the sample by racial/ethnic groups showed higher lead exposure in black women (eFigure 2A in the Supplement). In total, 229 mothers (15.9%) had RBC lead levels of at least 5.0 μg/dL. The median age of children at the time of the whole-blood lead measurement was 0.8 (IQR, 0.8-1.0) years. Sixty-six children (5.2%) had whole-blood lead levels of at least 5.0 μg/dL. Mothers with the highest RBC lead levels were older and multiparous, were more likely to be black and nonsmokers, had lower plasma folate levels, and were more likely to have prepregnancy OWO and diabetes (Table 1). Children whose mothers had RBC lead levels of at least 5.0 μg/dL had higher whole-blood lead levels in early childhood and were more likely to be OWO. Maternal RBC lead levels were positively associated with child whole-blood lead levels (eFigure 2B in the Supplement).

**Table 1.** Characteristics of 1442 Mother-Child Pairs Included in the Study

Characteristic	Maternal RBC Lead Level, No. (%)			*P* for Trend
	<2.0 μg/dL	2.0 to <5.0 μg/dL	≥5.0 μg/dL	
**Maternal**				
No.	513 (35.6)	700 (48.5)	229 (15.9)	NA
Maternal age, mean (SD), y	26.3 (6.0)	29.5 (6.5)	31.0 (5.8)	<.001
Educational level				
≤High school	328 (63.9)	464 (66.3)	144 (62.9)	.98
≥College	185 (36.1)	236 (33.7)	85 (37.1)	
Race/ethnicity				
Black	269 (52.4)	496 (70.9)	202 (88.2)	<.001
Hispanic	164 (32.0)	115 (16.4)	12 (5.2)	
Other	80 (15.6)	89 (12.7)	15 (6.6)	
Smoking status				
Nonsmoker	413 (80.5)	564 (80.6)	215 (93.9)	<.001
Smoker	100 (19.5)	136 (19.4)	14 (6.1)	
Parity				
Nulliparous	246 (48.0)	266 (38.0)	90 (39.3)	.004
Multiparous	267 (52.0)	434 (62.0)	139 (60.7)	
Prepregnancy BMI category				
Non-OWO mothers, BMI <25	261 (50.9)	320 (45.7)	100 (43.7)	.04
OWO mothers, BMI ≥25	252 (49.1)	380 (54.3)	129 (56.3)	
Gestational or preexisting diabetes	52 (10.1)	98 (14.0)	34 (14.8)	.04
Hypertensive disorder	71 (13.8)	115 (16.4)	30 (13.1)	.87
Cesarean delivery	186 (36.3)	257 (36.7)	78 (34.1)	.67
Maternal plasma folate level, nmol/L (95% CI)^a^	32.7 (31.0-34.4)	30.5 (29.1-31.9)	29.9 (27.6-32.4)	.04
**Child**				
Age, median (IQR), y	7.4 (5.5-9.9)	8.6 (6.0-10.6)	9.1 (6.7-11.0)	<.001
Sex				
Male	265 (51.7)	353 (50.4)	104 (45.4)	.15
Female	248 (48.3)	347 (49.6)	125 (54.6)	
Preterm birth	141 (27.5)	162 (23.1)	45 (19.7)	.01
Fetal growth				
AGA	412 (80.3)	539 (77.0)	185 (80.8)	.46
SGA	53 (10.3)	81 (11.6)	17 (7.4)	
LGA	48 (9.4)	80 (11.4)	27 (11.8)	
Breastfeeding status				
Formula exclusively	131 (25.5)	178 (25.4)	47 (20.5)	.37
Both formula and breastfed	342 (66.7)	455 (65.0)	167 (72.9)	
Breastfed exclusively	40 (7.8)	67 (9.6)	15 (6.6)	
OWO	191 (37.2)	311 (44.4)	110 (48.0)	.002
Postnatal lead level, μg/dL (95% CI)^a^	1.8 (1.7-1.8)	1.9 (1.9-2.0)	2.1 (2.0-2.3)	<.001

Abbreviations: AGA, appropriate for gestational age; BMI, body mass index (calculated as prepregnancy weight in kilograms divided by height in meters squared); IQR, interquartile range; LGA, large for gestational age; NA, not applicable; OWO, overweight or obesity; RBC, red blood cell; SGA, small for gestational age.

SI conversion factors: To convert lead level to micromoles per liter, multiply by 0.0483; folate level to nanomoles per liter, multiply by 2.266.

^a^ Data are presented as the geometric mean (n = 1201).

### Maternal RBC Lead Levels and Child BMI *z* Scores and OWO

**Figure 1.**
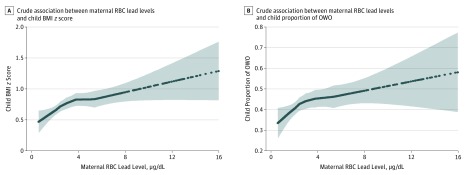
Smooth Plots of Maternal Red Blood Cell (RBC) Lead Levels and Child Body Mass Index (BMI) *z* Scores and Child Proportion of Overweight or Obesity (OWO) Because of the small sample size, the curves are truncated at 16 μg/L (n = 18). The curves (and 95% CIs [indicated by the shaded areas]) were derived from smoothing plots generated using the PROC LOESS step in SAS statistical software (version 9.4; SAS Institute Inc). To convert lead level to micromoles per liter, multiply by 0.0483.

Maternal RBC lead levels were positively associated with child BMI *z* scores (Figure 1A) and child proportion of OWO (Figure 1B). This association was similar across sex strata (eFigure 3 in the Supplement) and across child whole-blood lead levels (eFigure 4 in the Supplement). Children born to mothers who had RBC lead levels in the range of 2.0 to less than 5.0 μg/dL and at least 5.0 μg/dL had β coefficient (SE) increases in child BMI *z* scores of 0.19 (0.07) and 0.34 (0.10), respectively, and had odds ratios (ORs) for increased OWO risk compared with children whose mothers had RBC lead levels of less than 2.0 μg/dL of 1.35 (95% CI, 1.05-1.72) and 1.65 (95% CI, 1.18-2.32) after adjustment for potential confounders, including maternal educational level, race/ethnicity, smoking status, parity, diabetes, hypertensive disorder, preterm birth, fetal growth, and breastfeeding status (Table 2). The associations were consistent among non-OWO mothers and OWO mothers (Table 2).

**Table 2.** Individual and Combined Associations Between Maternal OWO Status and Maternal RBC Lead Levels, and Child BMI *z* Scores and Child OWO Risk (Age Range, 2-15 Years)^a^

Maternal Status by OWO and RBC Lead Level, μg/dL	Mother-Child Dyad, No.	Child BMI *z* Score			Child OWO		
		Mean (SD)	β (SE)	*P* Value	Child OWO, No. (%)	OR (95% CI)	*P* Value
Non-OWO mothers	681	0.40 (1.21)	1 [Reference]	NA	213 (31.3)	1 [Reference]	NA
OWO mothers	761	1.02 (1.15)	0.53 (0.06)	<.001	399 (52.4)	2.18 (1.74-2.74)	<.001
<2.0	513	0.57 (1.25)	1 [Reference]	NA	191 (37.2)	1 [Reference]	NA
2.0 to <5.0	700	0.78 (1.20)	0.19 (0.07)	.006	311 (44.4)	1.35 (1.05-1.72)	.02
≥5.0	229	0.92 (1.16)	0.34 (0.10)	<.001	110 (48.0)	1.65 (1.18-2.32)	.004
*P* for linear trend	NA	NA	NA	<.001	NA	NA	.01
**Stratified**							
Among non-OWO mothers							
<2.0	261	0.22 (1.17)	1 [Reference]	NA	65 (24.9)	1 [Reference]	NA
2.0 to <5.0	320	0.51 (1.24)	0.29 (0.10)	.005	113 (35.3)	1.65 (1.13-2.41)	.009
≥5.0	100	0.54 (1.17)	0.32 (0.15)	.03	35 (35.0)	1.81 (1.07-3.06)	.03
Among OWO mothers							
<2.0	252	0.95 (1.23)	1 [Reference]	NA	126 (50.0)	1 [Reference]	NA
2.0 to <5.0	380	1.00 (1.12)	0.08 (0.09)	.40	198 (52.1)	1.12 (0.80-1.57)	.52
≥5.0	129	1.21 (1.07)	0.32 (0.13)	.01	75 (58.1)	1.53 (0.96-2.44)	.07
**Combined**							
Non-OWO mothers							
<2.0	261	0.22 (1.17)	1 [Reference]	NA	65 (24.9)	1 [Reference]	NA
2.0 to <5.0	320	0.51 (1.24)	0.31 (0.10)	.001	113 (35.3)	1.71 (1.18-2.48)	.005
≥5.0	100	0.54 (1.17)	0.35 (0.14)	.01	35 (35.0)	1.79 (1.07-3.00)	.03
OWO mothers							
<2.0	252	0.95 (1.23)	0.65 (0.10)	<.001	126 (50.0)	2.78 (1.89-4.09)	<.001
2.0 to <5.0	380	1.00 (1.12)	0.72 (0.10)	<.001	198 (52.1)	3.08 (2.14-4.44)	<.001
≥5.0	129	1.21 (1.07)	0.94 (0.13	<.001	75 (58.1)	4.24 (2.64-6.82)	<.001

Abbreviations: BMI, body mass index (calculated as weight in kilograms divided by height in meters squared); NA, not applicable; OR, odds ratio; OWO, overweight or obesity; RBC, red blood cell.

SI conversion factor: To convert lead level to micromoles per liter, multiply by 0.0483.

^a^ Maternal OWO was defined as a BMI of 25 or higher. Analyses were performed by linear and logistic regression models for outcome child BMI *z* scores and child OWO, respectively. Adjusted for maternal educational level, race/ethnicity, smoking status, parity, diabetes, hypertensive disorder, preterm birth, fetal growth, and breastfeeding. *P* values for the interaction between maternal RBC lead levels and maternal OWO status were .29 for child BMI *z* scores and .41 for child OWO status.

A series of sensitivity analyses were performed, and the associations did not change materially after additional adjustment for child whole-blood lead level in early childhood (eTable 2 in the Supplement), maternal age (eTable 3 in the Supplement), cesarean delivery (eTable 4 in the Supplement), term births only (eTable 5 in the Supplement), and black children only (eTable 6 in the Supplement). The associations persisted from preschool age (2-5 years), to school age (6-9 years), to early adolescence (10-15 years) (eTable 7 in the Supplement). We also further adjusted for physical activity in a subset of children, and the results were similar (eTable 8 in the Supplement). The pattern of associations described above (ie, high maternal RBC lead levels associated with increased child BMI *z* scores and OWO risk) was also confirmed by a propensity score–matched analysis of 408 mother-child pairs, a method that could mimic a randomized trial and minimize uncontrolled confounders (eTable 9 in the Supplement).

### Combined or Additive Associations Between Maternal RBC Lead Levels and OWO and Child BMI *z* Scores and OWO Risk in Childhood

There was an additive association between maternal prepregnancy OWO higher RBC lead levels and OWO and child BMI *z* scores and OWO risk in childhood. Children of OWO mothers with RBC lead levels of at least 5.0 μg/dL had a β (SE) increase in child BMI *z* scores of 0.94 (0.13) and had an adjusted OR of 4.24 (95% CI, 2.64-6.82) for increased OWO risk compared with those whose non-OWO mothers had low RBC lead levels (<2.0 μg/dL) (Table 2).

### Child Whole-Blood Lead Levels, Child BMI *z* Scores, and OWO

There was no significant association between child whole-blood lead levels and child BMI *z* scores and OWO risk in childhood (eFigure 5 and eTable 10 in the Supplement). The associations between child whole-blood lead levels and OWO risk in childhood were not significant regardless of maternal OWO status (eTable 10 in the Supplement) and maternal RBC lead levels (eTable 11 in the Supplement). There was no interaction between maternal RBC lead levels and child whole-blood lead levels on child outcomes (child BMI *z* scores and OWO risk in childhood) (eFigure 4 and eTable 11 in the Supplement). Sensitivity analyses showed that child whole-blood lead levels were not significantly associated with child insulin and leptin levels regardless of maternal RBC lead levels (eTable 12 in the Supplement).

### Possible Mediation by Insulin and Leptin in Biological Pathways

Potential biological pathways were explored, and maternal RBC lead levels were positively associated with child plasma insulin and leptin levels in early childhood at a median age of 1.1 (IQR, 0.8-2.3) years in a subset (eFigure 6A and B in the Supplement). Mediation analysis showed that insulin and leptin mediated 15.4% (95% CI, 4.4%-41.6%; *P* = .02) and 23.3% (95% CI, 8.1%-51.3%; *P* = .004), respectively, of the association between maternal RBC lead levels and child OWO risk (eFigure 6C in the Supplement).

### Role of Folate in the Association Between Maternal RBC Lead Levels and Child OWO

**Figure 2.**
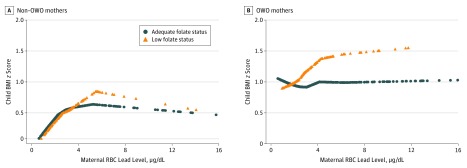
Smooth Plots of Maternal Red Blood Cell (RBC) Lead Levels and Child Body Mass Index (BMI) *z* Scores Stratified by Maternal Folate Status Crude association between maternal RBC lead concentration and offspring BMI *z* scores stratified by maternal folate status (low vs adequate level) among non–overweight or obese (non-OWO) mothers (A) and OWO mothers (B).Because of the small sample size, the curves are truncated at 16 μg/L (n = 6) (A) and at 16 μg/L (n = 8) (B). The curves were derived from smoothing plots generated using the PROC LOESS step in SAS statistical software (version 9.4; SAS Institute Inc). To convert lead level to micromoles per liter, multiply by 0.0483.

The association between maternal RBC lead levels and child BMI *z* scores differed according to maternal folate status among OWO mothers (Figure 2B). The association between maternal RBC lead levels and child BMI *z* scores remained in OWO mothers with low plasma folate levels but disappeared among OWO mothers with adequate folate status (Figure 2B) and was confirmed by regression analyses (Table 3). Children of OWO mothers with high RBC lead levels had a β (SE) of 0.31 (0.13) decrease in BMI *z* scores and 41% lower OWO risk (OR, 0.59; 95% CI, 0.36-0.95) if their mothers had adequate plasma folate levels (≥20.4 nmol/L) compared with their counterparts. A test of interaction between maternal RBC lead levels and plasma folate levels on child BMI *z* scores was not significant. Sensitivity analysis showed that maternal plasma folate levels were positively associated with maternal self-reported prenatal multivitamin (containing 800 μg of folic acid per tablet) intake in the second and third trimesters (eTable 13 in the Supplement). We observed that higher frequency of maternal self-reported prenatal multivitamin intake had similar protective consequences against intergenerational OWO risk (eTable 14 and eTable 15 in the Supplement).

**Table 3.** Role of Maternal Folate Status in the Association Between Maternal RBC Lead Levels and Child BMI *z* Scores and Child OWO Risk (Age Range, 2-15 Years) Stratified by Maternal OWO Status^a^

Maternal RBC Lead Level, μg/dL	Maternal Folate	Mother-Child Dyad, No.	Child BMI *z* Score			Child OWO		
			Mean (SD)	β (SE)	*P* Value	Child OWO, No. (%)	OR (95% CI)	*P* Value
Non-OWO mothers (n = 565)								
<2.0	Low	36	0.20 (1.23)	1 [Reference]	NA	10 (27.8)	1 [Reference]	NA
<2.0	Adequate	178	0.23 (1.19)	0.03 (0.22)	.89	44 (24.7)	0.86 (0.35-2.09)	.74
≥2.0	Low	86	0.56 (1.30)	1 [Reference]	NA	33 (38.4)	1 [Reference]	NA
≥2.0	Adequate	265	0.52 (1.18)	−0.01 (0.15)	.95	91 (34.3)	0.88 (0.51-1.51)	.64
OWO mothers (n = 644)								
<2.0	Low	37	0.94 (1.22)	1 [Reference]	NA	18 (48.6)	1 [Reference]	NA
<2.0	Adequate	161	0.97 (1.27)	0.08 (0.21)	.72	82 (50.9)	1.15 (0.52-2.51)	.73
≥2.0	Low	100	1.29 (1.12)	1 [Reference]	NA	63 (63.0)	1 [Reference]	NA
≥2.0	Adequate	346	0.97 (1.10)	−0.31 (0.13)	.02	175 (50.6)	0.59 (0.36-0.95)	.03

Abbreviations: BMI, body mass index (calculated as weight in kilograms divided by height in meters squared); NA, not applicable; OR, odds ratio; OWO, overweight or obesity; RBC, red blood cell.

SI conversion factor: To convert lead level to micromoles per liter, multiply by 0.0483.

^a^ Maternal OWO was defined as a BMI of 25 or higher. Analyses were restricted to those with available folate levels (n = 1209). Low folate status was defined as plasma folate levels of less than 20.4 nmol/L. Adequate folate status was defined as plasma folate levels of at least 20.4 nmol/L. Analyses were performed by linear and logistic regression models for outcome child BMI *z* scores and child OWO, respectively. Adjusted for maternal educational level, race/ethnicity, smoking status, parity, diabetes, hypertensive disorder, preterm birth, fetal growth, breastfeeding, and child early childhood blood lead level. Among OWO mothers, *P* values for the interaction between maternal RBC lead levels and maternal folate status were .07 for child BMI *z* scores and .17 for child OWO.

## Discussion

To our knowledge, this is the first large prospective birth cohort study to investigate the association of maternal exposure and early-life exposure to lead with OWO risk from preschool age to adolescence. When considered simultaneously, maternal lead exposure, rather than early childhood lead exposure, contributed to OWO risk in a dose-response fashion across multiple developmental stages (preschool age, school age, and early adolescence) and amplified intergenerational OWO risk (additively with maternal OWO). Furthermore, adequate maternal folate status mitigated the association between maternal lead exposure and child OWO, especially among children born to OWO mothers. These findings support the hypothesis that the obesity epidemic could be related to environmental chemical exposures in utero and raise the possibility that optimal maternal folate supplementation may help counteract the adverse effects of environmental lead exposure.

Our findings are consistent with an animal study,^16^ which provided strong evidence that maternal lead exposure increases body weight. Epidemiological studies of maternal lead exposure and childhood OWO are limited and inconsistent. Afeiche et al^17^ reported that a decrease in child weight over time up to age 5 years correlated with lead levels in maternal patella bone and was confined to girls, whereas maternal tibial lead levels were associated with a nonsignificant increased body weight among boys in adjusted models. In another study,^18^ maternal urine lead levels were not associated with child body weight at age 5 years. Inconsistencies across studies may be due to different measures of lead exposure (bone, urine, and blood) and variations in population demographics. In addition, some studies^29,30^ did not take into account maternal OWO status, an important risk factor for childhood obesity.

Our study found no significant association between lead exposure in early childhood and OWO in later life. Previous findings about exposure to lead in childhood and obesity are inconclusive. Two cross-sectional studies^31,32^ found that higher lead levels in blood and urine were associated with reduced OWO risk between ages 6 and 19 years, while another study^29^ observed that dentin lead levels were positively associated with BMI at age 7 years, which persisted to young adulthood. In adults, a study^33^ reported a positive association between higher blood lead levels and greater BMI and waist circumference. An animal study^34^ found that maternal lead exposure was associated with offspring obesity occurring in older age, but early postnatal exposure was not associated with the risk of obesity. Taken together, the inconsistency of the findings may be due to different study designs, sources of biological samples, and age groups, as well as varying ability to control for major confounding factors, including maternal lead exposure and OWO status. The present study is among the first studies to simultaneously examine maternal and early childhood lead levels and maternal OWO status in assessing childhood OWO risk.

Our findings are biologically plausible. The in utero period has been recognized as a critical developmental stage for obesity because of rapid cellular growth, differentiation, epigenome establishment,^35,36^ and metabolic programming.^36,37^ The fetus is particularly vulnerable to nutritional and environmental exposures.^38,39^ A previous study^40^ demonstrated a notable transplacental passage of maternal lead to the fetus in humans. Another study^41^ in humans revealed that maternal lead exposure was associated with altered DNA methylation in cord blood. An animal study^42^ showed that lead exposure can alter expression of methyltransferases and methyl cytosine–binding proteins, which regulate DNA methylation. Taken together, maternal lead exposure may change fetal metabolism by altered DNA methylation. Moreover, the present study showed that maternal lead exposure is associated with plasma insulin and leptin levels in early childhood, and insulin and leptin mediated the associations between maternal lead exposure and OWO risk by 15.4% and 23.3%, respectively. These results are supported by an animal study^16^ in which mice with maternal lead exposure displayed increased food intake and increased blood insulin levels.

While chelation treatment can be used in the setting of high-level lead exposure, effective interventions for reducing the adverse health consequences of low-level lead exposures are lacking. Previous work revealed that adequate maternal folate status may mitigate the intergenerational risk of obesity.^19^ The present study further revealed that adequate folate status reduced the detrimental consequences of lead exposure among OWO mothers. As one of the primary methyl group donors that maintain numerous cellular functions,^43,44^ folate has a critical role in placental development^45,46,47^ and fetal growth.^48^ While exact mechanisms require further exploration, folate supplementation has the potential to counteract the consequences of lead exposure. However, one-time measurement of plasma folate levels may or may not reflect long-term folate status, depending on the stability of intake of folic acid supplementation or foods enriched with folic acid over time. In our study, maternal plasma folate levels were positively associated with maternal self-reported prenatal multivitamin (containing 800 μg of folic acid per tablet) intake in the second and third trimesters. Therefore, maternal plasma folate levels at least partially reflect maternal folic acid intake during pregnancy. Furthermore, we found similar protective associations with plasma folate levels for higher frequency of self-reported prenatal multivitamin intake among OWO mothers, suggesting that adequate maternal folate status during mid-to-late pregnancy may have protective consequences.

### Strengths and Limitations

Our study has several strengths. We simultaneously assessed the association between prenatal and early childhood exposures to lead and child metabolic health and considered maternal prepregnancy OWO status. Because most blood lead was stored in RBCs, the hemodynamic changes inherent in pregnancy may potentially alter whole-blood lead levels. Therefore, assessment of RBC lead levels avoids the influence of hematocrit changes during pregnancy. Prior studies showed that lead in RBCs more accurately reflects the transplacental transfer of lead from the mother to the fetus compared with plasma samples.^40^ Lead level was assessed in our study in maternal RBCs obtained 1 to 3 days after delivery, a reasonable proxy of exposure in the third trimester because the RBC life span is approximately 120 days.^49^

Our study has some limitations. First, maternal lead exposure was only measured 1 time in our sample; therefore, we could not examine the consequences of lead exposure during early gestational periods. However, epidemiological studies have shown that the third trimester is a critical period for fetal adiposity development.^50^ Second, although a series of sensitivity analyses indicated that our findings in this study are robust and biologically plausible, we did not measure dietary intake and physical activity in all children. As such, we were unable to eliminate all potential residual confounding in our results. Third, given that we had maternal plasma folate levels at only 1 time point, it is difficult to identify the exact time window of pregnancy during which maternal folate status may offer protective consequences. Future studies should further explore trimester-specific protective associations of folate level. Fourth, our study was conducted in a predominantly urban low-income minority population in the United States; therefore, caution is needed in generalizing our findings to other populations with different characteristics.

## Conclusions

In this large, long-term prospective birth cohort study of a US urban low-income minority population, a significant dose-response association between maternal lead exposure and increased risk of child OWO was demonstrated. Our findings also indicate that maternal lead exposure may be associated with increased intergenerational OWO and that adequate maternal folate status may mitigate the OWO risk associated with maternal lead exposure. These results warrant additional investigation; if further confirmed, they raise the possibility that a combination of prenatal lead screening and optimal maternal folate nutrition may inform a new public health strategy to identify and decrease intergenerational lead toxic effects and OWO risk among US urban low-income populations, beginning in the most sensitive in utero developmental period.

## Notes

Supplement. **eFigure 1.** Flowchart of the Sample Included in the Analysis **eFigure 2.** Distribution of Maternal RBC Lead Levels in Total Sample and by Race/Ethnicity (Panel A) and Relationship Between Maternal RBC Lead and Child Blood Lead With 95% Confidence Interval (Panel B) **eFigure 3.** Smooth Plots of Maternal RBC Lead and Child BMI *z*-Score and OWO Stratified by Gender **eFigure 4.** Smooth Plots of Maternal RBC Lead and Child BMI *z*-Score and OWO Stratified by Child Blood Lead Levels **eFigure 5.** Association Between Maternal RBC Lead and Child Blood Lead Levels and Child BMI *z*-Score and Proportion of OWO **eFigure 6.** Maternal RBC Lead and Child Early Childhood Leptin z-Score (Panel A, n=822) and Child Early Childhood Insulin *z*-Score (Panel B, n=572); and Estimated Degree of Mediation by Leptin and Insulin on the Association Between Maternal RBC Lead and Child BMI *z* Score (Panel C) **eTable 1.** Comparison of Prenatal and Early Childhood Characteristics Between Included and Excluded Samples **eTable 2.** The Individual and Combined Associations of Maternal RBC-Lead Levels and Overweight or Obesity (OWO) Status on Child BMI *z*-Score and OWO Risk (Age Range: 2-15 Years), With Additional Adjustment for Child’s Blood Lead in Early Childhood **eTable 3.** The Individual and Combined Associations of Maternal Overweight or Obesity (OWO) Status and RBC Lead on Child BMI *z*-Score and OWO Risk (Age Range: 2-15 Years), With Additional Adjustment for Maternal Age **eTable 4.** The Individual and Combined Associations of Maternal RBC-Lead Levels and Overweight or Obesity (OWO) Status on Child BMI *z*-Score and OWO Risk (Age Range: 2-15 Years), With Additional Adjustment for Cesarean Section **eTable 5.** The Individual and Combined Associations of Maternal RBC-Lead Levels and Overweight or Obesity (OWO) Status on Child BMI *z*-Score and OWO Risk (Age Range: 2-15 Years), Among Term Births (n=1094) **eTable 6** The Individual and Combined Associations of Maternal RBC-Lead Levels and Overweight or Obesity (OWO) Status on Child BMI *z*-Score and OWO Risk (Age Range: 2-15 Years) Among Black Children (n=967) **eTable 7.** The Individual and Combined Associations of Maternal RBC-Lead Levels and Overweight or Obesity (OWO) Status on Child BMI *z*-Scores and OWO Risk, Stratified by Child Age Groups: 2-5, 6-9, and 10-15 Years **eTable 8.** The Individual and Combined Associations of Maternal RBC-Lead Levels and Overweight or Obesity (OWO) Status on Child BMI *z*-Score and OWO Risk (Age Range: 2-15 Years, n=704) Further Adjusted for Physical Activity **eTable 9.** Propensity Score–Based Matched Analysis for Comparison of Low Lead (<2 µg/dL) vs. High Lead (≥5 µg/dL) **eTable 10.** The Individual and Combined Associations of Maternal Overweight or Obesity (OWO) Status and Child Blood Lead Levels on Child BMI *z-*Score and OWO Risk (Age Range: 2-15 years, n=1271) **eTable 11.** The Individual and Combined Associations of Maternal RBC Lead and Child Blood Lead Levels on Child BMI *z*-Score and Overweight or Obesity (OWO) Risk (Age Range: 2-15 Years, n=1271) **eTable 12.** The Individual and Combined Associations of Maternal RBC Lead and Child Blood Lead Levels on Child Leptin and Insulin *z*-Scores **eTable 13.** Maternal Plasma Folate Levels According to Prenatal Vitamin Intake During 3 Trimesters **eTable 14.** Role of Maternal Folic Acid Intake During 2nd Trimester in the Associations of Maternal RBC-Lead Levels and Child BMI *z*-Score and Overweight or Obesity (OWO) Risk (Age Range: 2-15 Years), Stratified by Maternal OWO Status **eTable 15.** Role of Maternal Folic Acid Intake During 3rd Trimester in the Associations of Maternal RBC-Lead Levels and Child BMI *z-*Score and Overweight or Obesity (OWO) Risk (Age Range: 2-15 Years), Stratified by Maternal OWO Status
