# Supplementary material for: Association Between Maternal Exposure to Lead, Maternal Folate Status, and Intergenerational Risk of Childhood Overweight and Obesity
Source: JAMA Netw Open. 2019 Oct 2;2(10):e1912343. doi: 10.1001/jamanetworkopen.2019.12343 (PMC6777254; doi:10.1001/jamanetworkopen.2019.12343)
Supplement: Supplement. — eFigure 1. Flowchart of the Sample Included in the Analysis eFigure 2. Distribution of Maternal RBC Lead Levels in Total Sample and by Race/Ethnicity (Panel A) and Relationship Between Maternal RBC Lead and Child Blood Lead With 95% Confidence Interval (Panel B) eFigure 3. Smooth Plots of Maternal RBC Lead and Child BMI z-Score and OWO Stratified by Gender eFigure 4. Smooth Plots of Maternal RBC Lead and Child BMI z-Score and OWO Stratified by Child Blood Lead Levels eFigure 5. Association Between Maternal RBC Lead and Child Blood Lead Levels and Child BMI z-Score and Proportion of OWO eFigure 6. Maternal RBC Lead and Child Early Childhood Leptin z-Score (Panel A, n=822) and Child Early Childhood Insulin z-Score (Panel B, n=572); and Estimated Degree of Mediation by Leptin and Insulin on the Association Between Maternal RBC Lead and Child BMI z Score (Panel C) eTable 1. Comparison of Prenatal and Early Childhood Characteristics Between Included and Excluded Samples eTable 2. The Individual and Combined Associations of Maternal RBC-Lead Levels and Overweight or Obesity (OWO) Status on Child BMI z-Score and OWO Risk (Age Range: 2-15 Years), With Additional Adjustment for Child’s Blood Lead in Early Childhood eTable 3. The Individual and Combined Associations of Maternal Overweight or Obesity (OWO) Status and RBC Lead on Child BMI z-Score and OWO Risk (Age Range: 2-15 Years), With Additional Adjustment for Maternal Age eTable 4. The Individual and Combined Associations of Maternal RBC-Lead Levels and Overweight or Obesity (OWO) Status on Child BMI z-Score and OWO Risk (Age Range: 2-15 Years), With Additional Adjustment for Cesarean Section eTable 5. The Individual and Combined Associations of Maternal RBC-Lead Levels and Overweight or Obesity (OWO) Status on Child BMI z-Score and OWO Risk (Age Range: 2-15 Years), Among Term Births (n=1094) eTable 6 The Individual and Combined Associations of Maternal RBC-Lead Levels and Overweight or Obesity (OWO) Status on Child BMI z [file jamanetwopen-2-e1912343-s001.pdf]

Supplementary Online Content

Wang G, DiBari J, Bind E, et al. Association between maternal exposure to lead, maternal folate status, and intergenerational risk of childhood overweight and obesity. *JAMA Netw Open*. 2019;2(10):e1912343. doi:10.1001/jamanetworkopen.2019.12343

eFigure 1. Flowchart of the Sample Included in the Analysis

eFigure 2. Distribution of Maternal RBC Lead Levels in Total Sample and by Race/Ethnicity (Panel A) and Relationship Between Maternal RBC Lead and Child Blood Lead With 95% Confidence Interval (Panel B)

eFigure 3. Smooth Plots of Maternal RBC Lead and Child BMI *z*-Score and OWO Stratified by Gender

eFigure 4. Smooth Plots of Maternal RBC Lead and Child BMI *z*-Score and OWO Stratified by Child Blood Lead Levels

eFigure 5. Association Between Maternal RBC Lead and Child Blood Lead Levels and Child BMI *z*-Score and Proportion of OWO

eFigure 6. Maternal RBC Lead and Child Early Childhood Leptin z-Score (Panel A, n=822) and Child Early Childhood Insulin *z*-Score (Panel B, n=572); and Estimated Degree of Mediation by Leptin and Insulin on the Association Between Maternal RBC Lead and Child BMI *z* Score (Panel C)

eTable 1. Comparison of Prenatal and Early Childhood Characteristics Between Included and Excluded Samples

eTable 2. The Individual and Combined Associations of Maternal RBC-Lead Levels and Overweight or Obesity (OWO) Status on Child BMI *z*-Score and OWO Risk (Age Range: 2-15 Years), With Additional Adjustment for Child's Blood Lead in Early Childhood

eTable 3. The Individual and Combined Associations of Maternal Overweight or Obesity (OWO) Status and RBC Lead on Child BMI *z*-Score and OWO Risk (Age Range: 2-15 Years), With Additional Adjustment for Maternal Age

eTable 4. The Individual and Combined Associations of Maternal RBC-Lead Levels and Overweight or Obesity (OWO) Status on Child BMI *z*-Score and OWO Risk (Age Range: 2-15 Years), With Additional Adjustment for Cesarean Section

eTable 5. The Individual and Combined Associations of Maternal RBC-Lead Levels and Overweight or Obesity (OWO) Status on Child BMI *z*-Score and OWO Risk (Age Range: 2-15 Years), Among Term Births (n=1094)

eTable 6 The Individual and Combined Associations of Maternal RBC-Lead Levels and Overweight or Obesity (OWO) Status on Child BMI *z*-Score and OWO Risk (Age Range: 2-15 Years) Among Black Children (n=967)

eTable 7. The Individual and Combined Associations of Maternal RBC-Lead Levels and Overweight or Obesity (OWO) Status on Child BMI *z*-Scores and OWO Risk, Stratified by Child Age Groups: 2-5, 6-9, and 10-15 Years

eTable 8. The Individual and Combined Associations of Maternal RBC-Lead Levels and Overweight or Obesity (OWO) Status on Child BMI *z*-Score and OWO Risk (Age Range: 2-15 Years, n=704) Further Adjusted for Physical Activity

eTable 9. Propensity Score–Based Matched Analysis for Comparison of Low Lead (<2 µg/dL) vs. High Lead (≥5 µg/dL)

eTable 10. The Individual and Combined Associations of Maternal Overweight or Obesity (OWO) Status and Child Blood Lead Levels on Child BMI *z*-Score and OWO Risk (Age Range: 2-15 years, n=1271)

eTable 11. The Individual and Combined Associations of Maternal RBC Lead and Child Blood Lead Levels on Child BMI *z*-Score and Overweight or Obesity (OWO) Risk (Age Range: 2-15 Years, n=1271)

eTable 12. The Individual and Combined Associations of Maternal RBC Lead and Child Blood Lead Levels on Child Leptin and Insulin *z*-Scores

eTable 13. Maternal Plasma Folate Levels According to Prenatal Vitamin Intake During 3 Trimesters

eTable 14. Role of Maternal Folic Acid Intake During 2nd Trimester in the Associations of Maternal RBC-Lead Levels and Child BMI *z*-Score and Overweight or Obesity (OWO) Risk (Age Range: 2-15 Years), Stratified by Maternal OWO Status

eTable 15. Role of Maternal Folic Acid Intake During 3rd Trimester in the Associations of Maternal RBC-Lead Levels and Child BMI *z*-Score and Overweight or Obesity (OWO) Risk (Age Range: 2-15 Years), Stratified by Maternal OWO Status

This supplementary material has been provided by the authors to give readers additional information about their work.

© 2019 Wang G et al. *JAMA Network Open*.

eFigure 1. Flowchart of the sample included in the analysis

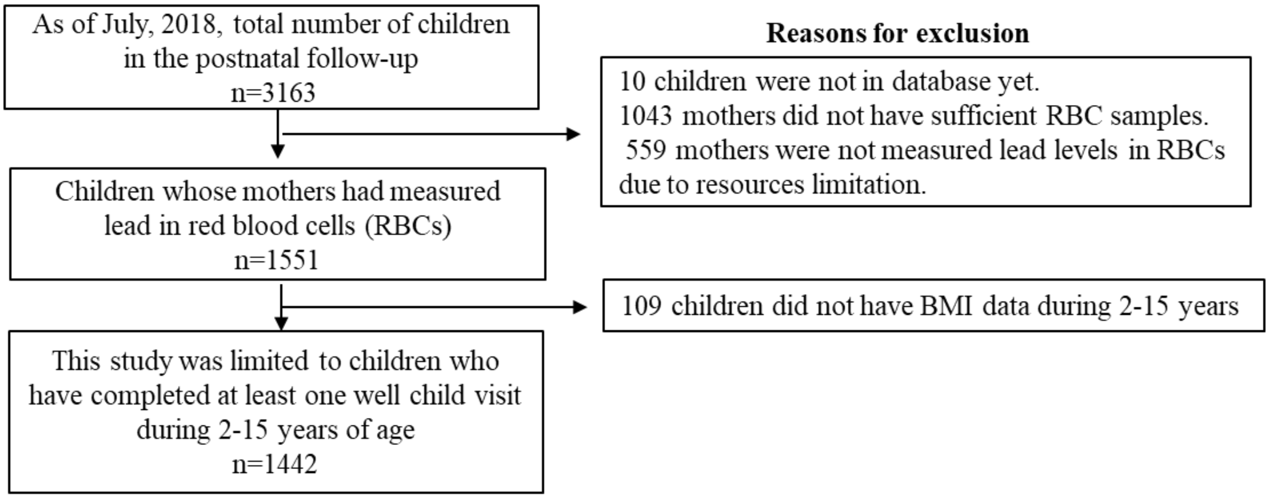

eFigure 2. Distribution of maternal RBC lead levels in total sample and by race/ethnicity (Panel A); and relationship between maternal RBC lead and child blood lead with 95% conference interval (Panel B).

A.

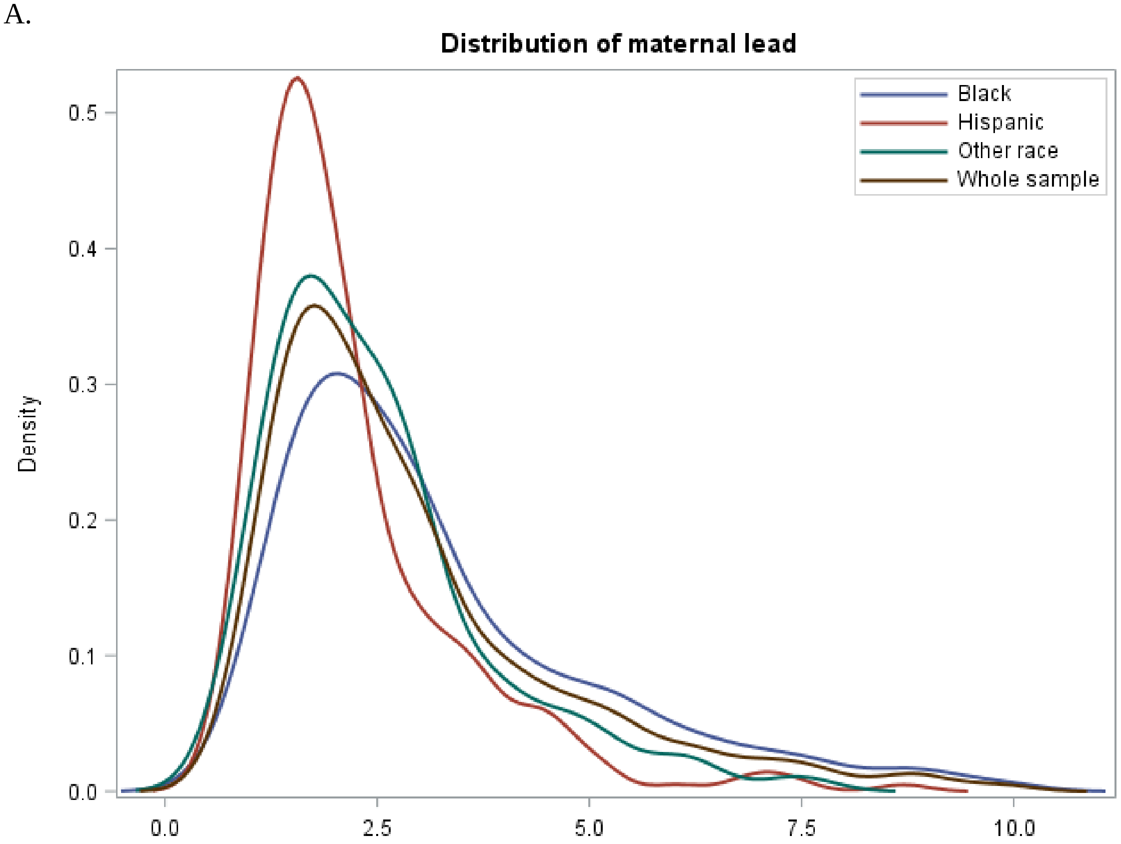

Maternal lead was truncated by 10µg/dL, 63 mothers had lead levels >10µg/dL.

B.

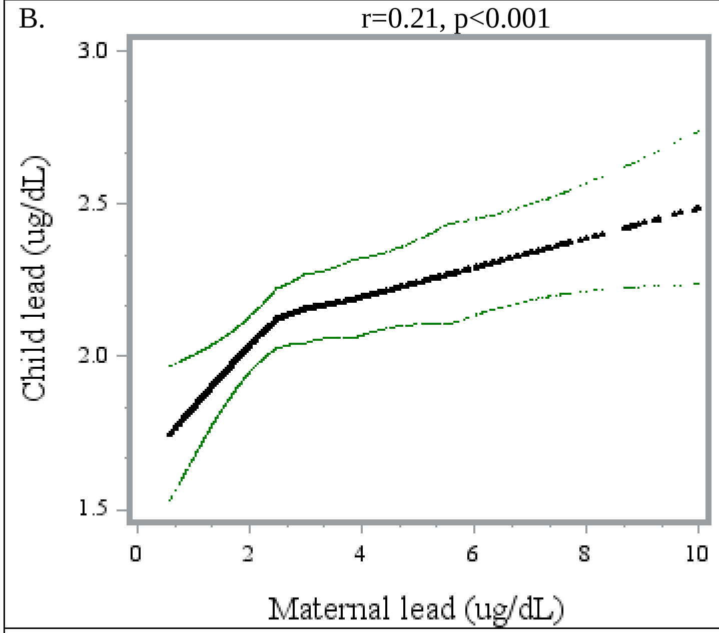

Green lines represent 95%CI

eFigure 3. Smooth plots of maternal RBC lead and child BMI z-score and OWO stratified by gender.

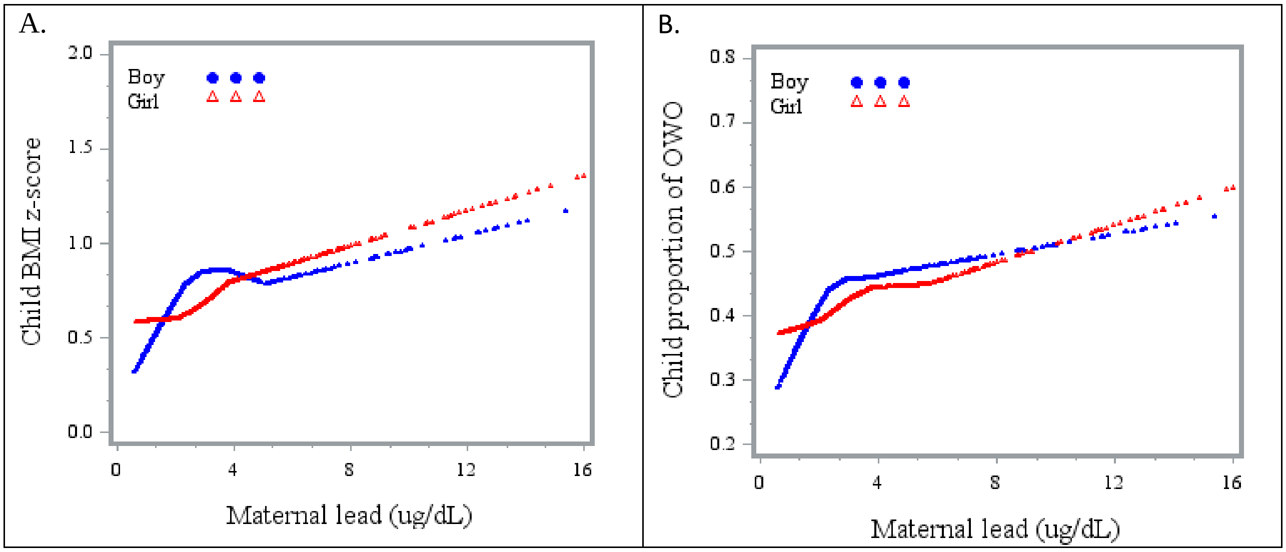

eFigure 4. Smooth plots of maternal RBC lead and child BMI z-score and OWO stratified by child blood lead levels.

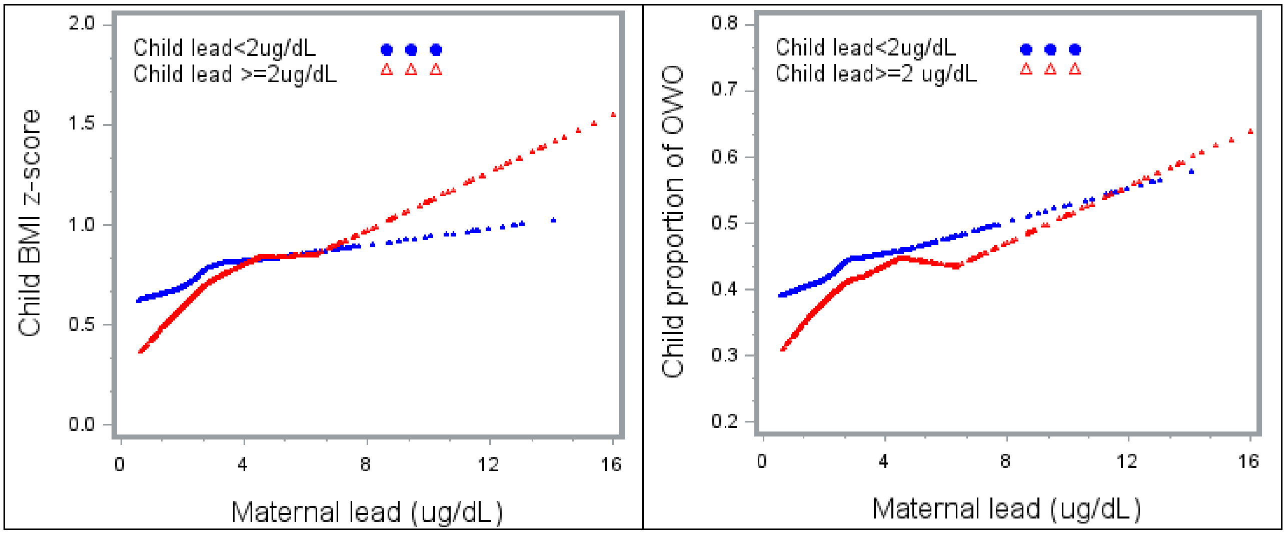

© 2019 Wang G et al. *JAMA Network Open*.

eFigure 5. Association between maternal RBC lead and child blood lead levels and child BMI z-score and proportion of OWO.

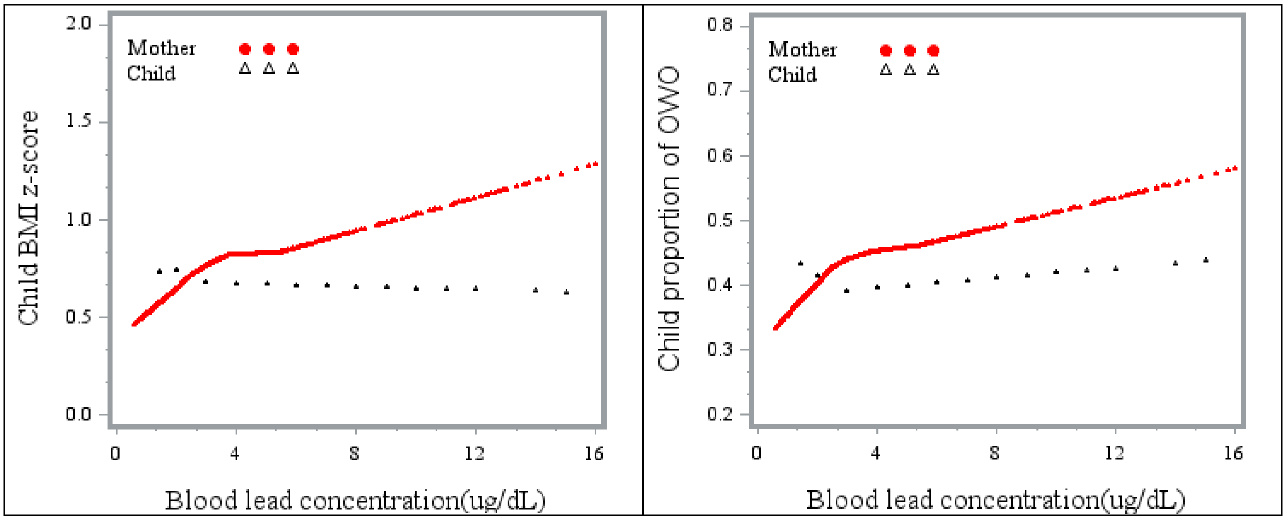

eFigure 6. Maternal RBC lead and child early childhood leptin z-score (Panel A, n=822); and child early childhood insulin z-score (Panel B, n=572); and estimated degree of mediation by leptin and insulin on the association between maternal RBC lead and child BMI z-score (Panel C).

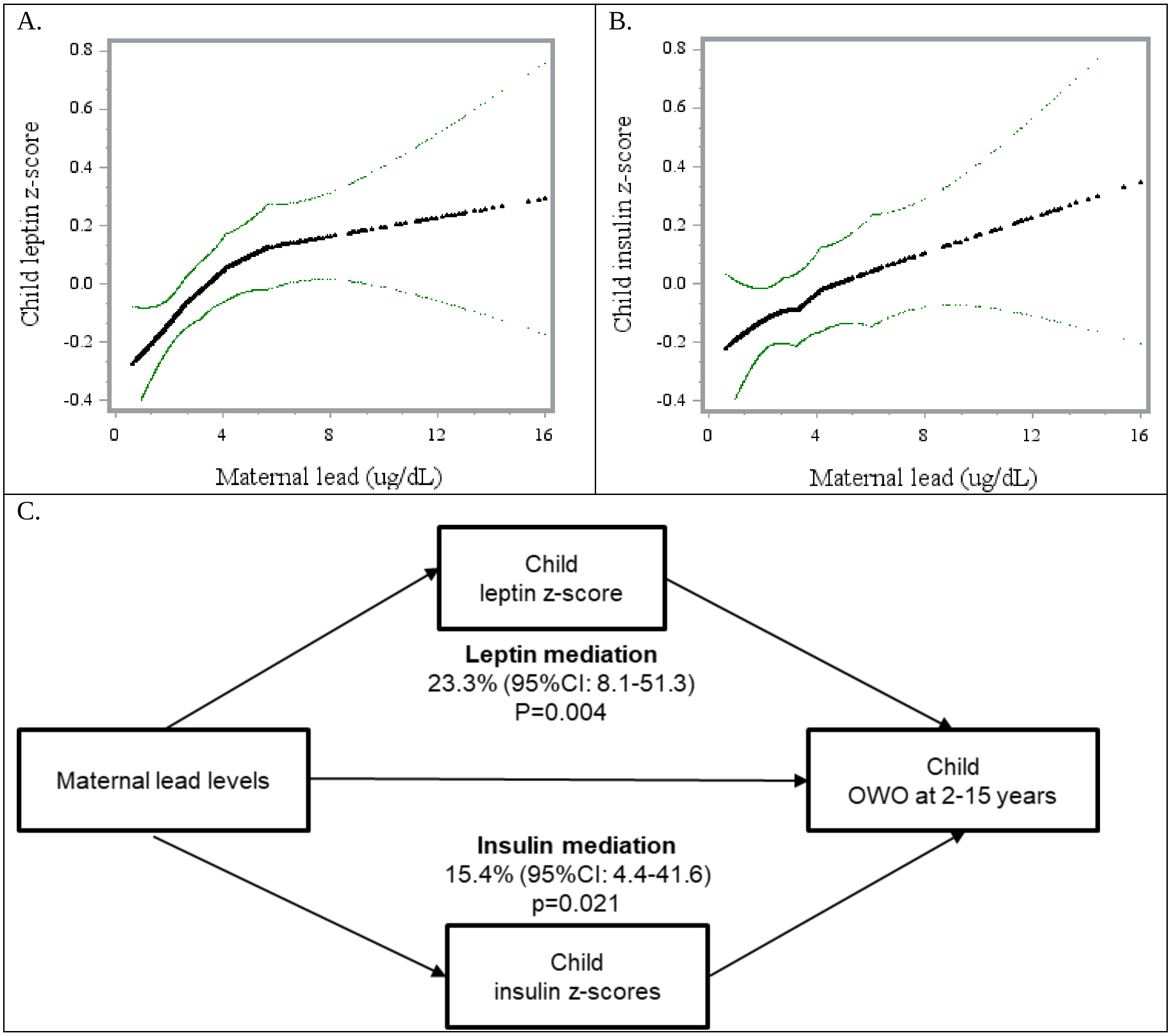

eTable 1. Comparison of the prenatal and early childhood characteristics between included and excluded samples

	Participants included in the study	Participants excluded in the study	P value
No.	1442	1711	
Maternal age (years)	28.6±6.5	28.5±6.5	0.741
Education			0.692
High school and below	936(64.9)	1099(64.2)	
College or above	506(35.1)	612(35.8)	
Race/ethnicity			0.002
Black	967(67.1)	1044(61.0)	
Hispanic	291(20.2)	411(24.0)	
Other	184(12.8)	256(15.0)	
Parity			0.363
Nulliparous	603(41.8)	743(43.4)	
Multiparous	839(58.2)	968(56.6)	
Smoking			0.017
Never	1192(82.7)	1381(80.7)	
Quitter	119(8.2)	123(7.2)	
Continuous	131(9.1)	207(12.1)	
Prepregnancy BMI categories			0.160
Lean	681(47.2)	851(49.7)	
OWO	761(52.8)	860(50.3)	
Diabetes status			0.476
Non-diabetes	1258(87.2)	1507(88.1)	
Gestational/preexisting diabetes	184(12.8)	204(11.9)	
Hypertensive disorder			0.659
No	1226(85.0)	1445(84.5)	
Yes	216(15.0)	266(15.5)	
Mode of delivery			0.653
Vaginal	921(63.9)	1106(64.6)	
Cesarean section	521(36.1)	605(35.4)	
Child's gender			0.736
Boy	722(50.1)	867(50.7)	
Girl	720(49.9)	844(49.3)	

eTable 2. The individual and combined associations of maternal RBC-lead levels and overweight or obesity (OWO) status on child BMI z-score and OWO risk (age range: 2-15 years), with additional adjustment for child's blood lead in early childhood.

Maternal status			Child BMI z-score				Child OWO			
**BMI**	**Lead (μg/dL)**	n	mean±SD	β	se	p	Case, n (%)	OR	95%CI	p
	<2	513	0.57±1.25	ref			191(37.2)	1.00		
	2-4	700	0.78±1.20	0.19	0.07	0.007	311(44.4)	1.36	1.06-1.73	0.015
	≥5	229	0.92±1.16	0.34	0.10	<0.001	110(48.0)	1.68	1.19-2.36	0.003
P for linear trend						<0.001				0.009
Combined										
Non-OWO	<2	261	0.22±1.17	ref			65(24.9)	1.00		
	2-4	320	0.51±1.24	0.31	0.10	0.001	113(35.3)	1.72	1.19-2.50	0.004
	≥5	100	0.54±1.17	0.34	0.14	0.013	35(35.0)	1.81	1.08-3.03	0.025
OWO	<2	252	0.95±1.23	0.65	0.10	<0.001	126(50.0)	2.76	1.87-4.06	<0.001
	2-4	380	1.00±1.12	0.71	0.10	<0.001	198(52.1)	3.08	2.13-4.44	<0.001
	≥5	129	1.21±1.07	0.93	0.13	<0.001	75(58.1)	4.27	2.65-6.89	<0.001

Abbreviation: BMI, body mass index; OWO, overweight or obesity.
Analyses were performed by linear and logistic regression models for outcome BMI z-scores and OWO, respectively.
Adjusted for maternal education, parity, smoking, race, diabetes, hypertensive disorders, preterm, fetal growth, breastfeeding, and child early childhood blood lead.

eTable 3. The individual and combined associations of maternal RBC-lead levels and overweight or obesity (OWO) status on child BMI z-score and OWO risk (age range: 2-15 years), with additional adjustment for maternal age.

Maternal status			Child BMI z-score				Child OWO			
**BMI**	**Lead (μg/dL)**	n	mean±SD	β	se	p	Case, n (%)	OR	95%CI	p
	<2	513	0.57±1.25	ref			191(37.2)	1.00		
	2-4	700	0.78±1.20	0.16	0.07	0.022	311(44.4)	1.30	1.01-1.68	0.041
	≥5	229	0.92±1.16	0.29	0.10	0.004	110(48.0)	1.58	1.10-2.25	0.012
P for linear trend						0.003				0.038
Combined										
Non-OWO	<2	261	0.22±1.17	ref			65(24.9)	1.00		
	2-4	320	0.51±1.24	0.30	0.10	0.002	113(35.3)	1.70	1.17-2.48	0.006
	≥5	100	0.54±1.17	0.31	0.14	0.025	35(35.0)	1.72	1.02-2.91	0.044
OWO	<2	252	0.95±1.23	0.63	0.10	<0.001	126(50.0)	2.69	1.83-3.97	<0.001
	2-4	380	1.00±1.12	0.67	0.10	<0.001	198(52.1)	2.88	1.98-4.19	<0.001
	≥5	129	1.21±1.07	0.89	0.13	<0.001	75(58.1)	4.02	2.46-6.58	<0.001

Abbreviation: BMI, body mass index; OWO, overweight or obesity.
Analyses were performed by linear and logistic regression models for outcome BMI z-scores and OWO, respectively.
Adjusted for maternal education, parity, smoking, race, diabetes, hypertensive disorders, preterm, fetal growth, breastfeeding, and maternal age.

eTable 4. The individual and combined associations of maternal RBC-lead levels and overweight or obesity (OWO) status on child BMI z-score and OWO risk (age range: 2-15 years), with additional adjustment for Cesarean section.

**Maternal status**			**Child BMI z-score**				**Child OWO**			
**BMI**	**Lead (μg/dL)**	n	mean±SD	β	se	p	Case, n (%)	OR	95%CI	p
	<2	513	0.57±1.25	ref			191(37.2)	1.00		
	2-4	700	0.78±1.20	0.19	0.07	0.006	311(44.4)	1.36	1.06-1.75	0.014
	≥5	229	0.92±1.16	0.34	0.10	<0.001	110(48.0)	1.70	1.20-2.40	0.003
P for linear trend						<0.001				0.007
Combined										
Non-OWO	<2	261	0.22±1.17	ref			65(24.9)	1.00		
	2-4	320	0.51±1.24	0.31	0.10	0.001	113(35.3)	1.75	1.20-2.54	0.004
	≥5	100	0.54±1.17	0.34	0.14	0.014	35(35.0)	1.80	1.07-3.03	0.030
OWO	<2	252	0.95±1.23	0.64	0.10	<0.001	126(50.0)	2.70	1.83-3.98	<0.001
	2-4	380	1.00±1.12	0.70	0.10	<0.001	198(52.1)	3.00	2.08-4.34	<0.001
	≥5	129	1.21±1.07	0.93	0.13	<0.001	75(58.1)	4.28	2.65-6.92	<0.001

Abbreviation: BMI, body mass index; OWO, overweight or obesity.
Analyses were performed by linear and logistic regression models for outcome BMI z-scores and OWO, respectively.
Adjusted for maternal education, parity, smoking, race, diabetes, hypertensive disorders, preterm, fetal growth, breastfeeding, and cesarean section.

eTable 5. The individual and combined associations of maternal RBC-lead levels and overweight or obesity (OWO) status on child BMI z-score and OWO risk (age range: 2-15 years) among term births (n=1094).

**Maternal status**			**Child BMI z-score**				**Child OWO**			
**BMI**	**Lead (μg/dL)**	n	mean±SD	β	Se	p	Case, n (%)	OR	95%CI	p
	<2	372	0.57±1.21	ref			136(36.6)	1.00		
	2-4	538	0.76±1.15	0.14	0.08	0.078	238(44.2)	1.29	0.97-1.72	0.082
	≥5	184	0.99±1.16	0.35	0.11	0.002	93(50.5)	1.76	1.18-2.61	0.005
P for linear trend						0.001				0.018
Combined										
Non-OWO	<2	200	0.22±1.18	ref			50(25.0)	1.00		
	2-4	247	0.48±1.18	0.24	0.11	0.025	83(33.6)	1.49	0.97-2.28	0.068
	≥5	79	0.64±1.18	0.36	0.15	0.019	30(38.0)	1.87	1.04-3.36	0.036
OWO	<2	172	0.97±1.12	0.63	0.12	<0.001	86(50.0)	2.60	1.65-4.10	<0.001
	2-4	291	1.00±1.08	0.63	0.11	<0.001	155(53.3)	2.87	1.89-4.38	<0.001
	≥5	105	1.25±1.07	0.89	0.14	<0.001	63(60.0)	4.08	2.37-7.01	<0.001

Abbreviation: BMI, body mass index; OWO, overweight or obesity.
Analyses were performed by linear and logistic regression models for outcome BMI z-scores and OWO, respectively.
Adjusted for maternal education, parity, smoking, race, diabetes, hypertensive disorders, preterm, fetal growth, breastfeeding.

eTable 6. The individual and combined associations of maternal RBC-lead levels and overweight or obesity (OWO) status on child BMI z-score and OWO risk (age range: 2-15 years) among Black children (n=967).

**Maternal status**			**Child BMI z-score**				**Child OWO**			
**BMI**	**Lead (μg/dL)**	n	mean±SD	β	Se	p	Case, n (%)	OR	95%CI	p
	<2	269	0.52±1.26	ref			97(36.1)	1.00		
	2-4	496	0.76±1.17	0.22	0.09	0.014	216(43.6)	1.31	0.95-1.80	0.095
	≥5	202	0.98±1.16	0.44	0.11	<0.001	99(49.0)	1.74	1.17-2.58	0.006
P for linear trend						<0.001				0.019
Combined										
Non-OWO	<2	131	0.12±1.20	ref			30(22.9)	1.00		
	2-4	210	0.48±1.19	0.36	0.13	0.005	68(32.4)	1.57	0.95-2.61	0.079
	≥5	86	0.62±1.16	0.49	0.16	0.002	32(37.2)	2.09	1.13-3.86	0.019
OWO	<2	138	0.90±1.21	0.72	0.14	<0.001	67(48.6)	2.96	1.72-5.10	<0.001
	2-4	286	0.97±1.12	0.77	0.12	<0.001	148(51.8)	3.26	2.01-5.30	<0.001
	≥5	116	1.25±1.09	1.04	0.15	<0.001	67(57.8)	4.34	2.45-7.67	<0.001

Abbreviation: BMI, body mass index; OWO, overweight or obesity.
Analyses were performed by linear and logistic regression models for outcome BMI z-scores and OWO, respectively.
Adjusted for maternal education, parity, smoking, race, diabetes, hypertensive disorders, preterm, fetal growth, breastfeeding.

eTable 7. The individual and combined associations of maternal RBC-lead levels and overweight or obesity (OWO) status on child BMI z-scores and OWO risk, stratified by child age groups: 2-5, 6-9, and 10-15 years.

Maternal status			Child BMI z-score				Child OWO			
**BMI**	**Lead (ug/dl)**	n	mean±SD	β	Se	p	Case, n (%)	OR	95%CI	p
				**Age 2-5 years (n=1395)**						
	<2	495	0.44±1.38	ref			163(32.9)	1.00		
	2-4	677	0.67±1.28	0.22	0.08	0.004	277(40.9)	1.42	1.10-1.83	0.008
	≥5	223	0.83±1.26	0.38	0.11	<0.001	109(48.9)	2.02	1.42-2.87	<0.001
Linear	P trend					<0.001				<0.001
**Combined**										
Non-OWO	<2	252	0.04±1.26	ref			53(21.0)	1.00		
	2-4	313	0.45±1.30	0.45	0.11	<0.001	111(35.5)	2.18	1.47-3.23	<0.001
	≥5	99	0.49±1.26	0.47	0.15	0.002	37(37.4)	2.36	1.39-4.01	0.002
OWO	<2	243	0.86±1.38	0.75	0.11	<0.001	110(45.3)	2.91	1.93-4.39	<0.001
	2-4	364	0.85±1.24	0.75	0.11	<0.001	166 (45.6)	2.94	1.99-4.34	<0.001
	≥5	124	1.09±1.21	1.00	0.14	<0.001	72(58.1)	5.14	3.12-8.46	<0.001
				**Age 6-9 years (n=1030)**						
	<2	349	0.69±1.23	ref			152(43.6)	1.00		
	2-4	508	0.85±1.16	0.15	0.08	0.067	238(46.9)	1.16	0.87-1.56	0.312
	≥5	173	0.96±1.15	0.26	0.11	0.023	86(49.7)	1.37	0.92-2.04	0.124
linear	P trend					0.014				0.256
**Combined**										
Non-OWO	<2	171	0.34±1.17	ref			51(29.8)	1.00		
	2-4	218	0.53±1.16	0.20	0.12	0.085	81(37.2)	1.46	0.94-2.26	0.095
	≥5	74	0.58±1.20	0.22	0.16	0.146	27(36.5)	1.46	0.80-2.66	0.215
OWO	<2	178	1.02±1.21	0.56	0.12	<0.001	101(56.7)	2.64	1.67-4.18	<0.001
	2-4	290	1.09±1.11	0.64	0.11	<0.001	157 (54.1)	2.50	1.63-3.83	<0.001
	≥5	99	1.25±1.04	0.81	0.15	<0.001	59(59.6)	3.35	1.92-5.83	<0.001
				**Age 10-15 years (n=449)**						
	<2	122	0.75±1.18	ref			49(40.2)	1.00		
	2-4	232	0.88±1.18	0.08	0.13	0.529	112(48.3)	1.27	0.79-2.06	0.322
	≥5	95	1.09±0.98	0.35	0.16	0.029	53(55.8)	2.04	1.12-3.72	0.021
linear	P trend					0.028				0.039
**Combined**										
Non-OWO	<2	58	0.46±1.02	ref			17(29.3)	1.00		
	2-4	100	0.43±1.23	-0.04	0.18	0.825	33(33.0)	1.14	0.54-2.38	0.733
	≥5	37	0.78±1.01	0.37	0.23	0.112	16(43.2)	2.14	0.85-5.37	0.105
OWO	<2	64	1.00±1.26	0.46	0.20	0.020	32(50.0)	2.17	0.98-4.82	0.057
	2-4	132	1.22±1.02	0.63	0.18	<0.001	79 (59.8)	3.03	1.48-6.21	0.003
	≥5	58	1.28±0.92	0.77	0.21	<0.001	37(63.8)	4.20	1.80-9.79	<0.001

Abbreviation: BMI, body mass index; OWO, overweight or obesity.
Analyses were performed by linear and logistic regression models for outcome BMI z-scores and OWO, respectively.
Adjusted for maternal education, parity, smoking, race, diabetes, hypertensive disorders, preterm, fetal growth, breastfeeding.

eTable 8. The individual and combined associations of maternal RBC-lead levels and overweight or obesity (OWO) status on child BMI z-score and OWO risk (age range: 2-15 years, n=704) further adjusted for physical activity.

**Maternal status**			**Offspring BMI z-score**				**Offspring OWO**			
**BMI**	**Lead (μg/dL)**	n	mean±SD	β	se	P	Case, n (%)	OR	95%CI	p
non-OWO		318	0.41±1.21	ref			101(31.8)	ref		
OWO		386	1.09±1.16	0.57	0.09	<0.001	213 (55.2)	2.35	1.69-3.28	<0.001
	<2	234	0.68±1.30	ref			94(40.2)	1.00		
	2-4	357	0.80±1.20	0.09	0.10	0.372	161(45.1)	1.21	0.85-1.72	0.298
	≥5	113	0.97±1.16	0.27	0.14	0.058	59(52.2)	1.64	1.01-2.68	0.046
P for linear trend						0.066				0.050
**Combined**										
non-OWO	<2	110	0.27±1.22	ref			32(29.1)	1.00		
	2-4	157	0.44±1.22	0.18	0.15	0.205	50(31.9)	1.16	0.67-2.00	0.599
	≥5	51	0.61±1.16	0.36	0.20	0.076	19(37.3)	1.56	0.75-3.24	0.263
OWO	<2	124	1.04±1.26	0.67	0.16	<0.001	62(50.0)	2.17	1.23-3.83	0.008
	2-4	200	1.07±1.12	0.71	0.15	<0.001	111(55.5)	2.84	1.66-4.86	<0.001
	≥5	62	1.27±1.07	0.93	0.19	<0.001	40(64.5)	4.28	2.10-8.71	<0.001

Abbreviation: BMI, body mass index; OWO, overweight or obesity.
Analyses were performed by linear and logistic regression models for outcome BMI z-scores and OWO, respectively.
Adjusted for maternal education, parity, smoking, race/ethnicity, diabetes, hypertensive disorders, preterm, fetal growth, breastfeeding, and physical activity.

eTable 9. Propensity score–based matched analysis for comparison of low lead (<2µg/dL) vs. high lead (≥5µg/dL)

Variable	Maternal low lead <2µg/dL	Maternal high lead ≥5µg/dL	SMD	P value
N	204	204		
Maternal lead levels, median(IQR),µg/dL	1.54(1.34, 1.75)	7.24(5.68, 10.30)		<0.001
Odds ratio	reference	1.26(1.03-1.54)		0.022
OWO rate (%)	76(37.25)	99(48.53)	0.172	0.021
Child BMI z-score	0.61±1.23	0.92±1.16	0.205	0.009
**Maternal characteristic**				
Race (%)			0.084	0.704
Black	177(86.76)	177(86.76)		
Hispanic	9(4.41)	12(5.88)		
Other	18(8.82)	15(7.35)		
Education (%)			0.000	0.760
High school and below	124(60.78)	127(62.25)		
College and above	80(39.22)	77(37.75)		
Maternal smoking			0.038	0.704
Never	188(92.16)	190(93.14)		
Continuous	16(7.84)	14(6.86)		
Parity			0.080	0.425
Nulliparous	94(46.08)	86(42.16)		
Multiparous	110(53.92)	118(57.84)		
BMI categories			0.080	0.372
Lean	100(49.02)	91(44.61)		
OWO	104(50.98)	113(55.39)		
Maternal diabetes (%)			-0.031	0.454
No	181(88.73)	176(86.27)		
Yes	23(11.27)	28(13.73)		
Hypertensive disorder			-0.014	0.777
No	174(85.29)	176(86.27)		
Yes	30(14.71)	28(13.73)		
Mode of delivery			0.031	0.604
Vaginal	135(66.18)	130(63.73)		
Cesarean section	69(33.82)	74(36.27)		
**Child's characteristic**				
Child's age, mean±SD, years	8.30±3.05	8.59±3.39	0.033	0.373
Gender (%)			0.040	0.843
Boy	93(45.59)	95(46.57)		
Girl	111(54.41)	109(53.43)		
Preterm	39(19.12)	42(20.59)	0.037	0.710
Fetal growth			0.048	0.973
Appropriate for gestational age	167(81.86)	167(81.86)		
Small for gestational age	17(8.33)	16(7.84)		
Large for gestational age	20(9.80)	21(10.29)		
Breastfeeding, n(%)			0.102	0.803
Formula	47(23.04)	45(22.06)		
Breastfeeding	10(4.90)	13(6.37)		
Both	147(72.06)	146(71.57)		

Abbreviation: BMI, body mass index (calculated as weight in kilograms divided by height in meters squared); OWO, overweight or obesity; SMD, Standardized Mean Difference.

Analysis: We applied a non-parismonious multivariable logistic regression model to calculate propensity scores for low lead (<2µg/dL) and. high lead (≥5µg/dL), using the following covariates: maternal education, race, smoking status, parity, diabetes status, hypertensive disorder, child's age, gender, preterm, fetal growth, and breastfeeding.

eTable 10. The individual and combined associations of maternal overweight or obesity (OWO) status and child blood lead levels on child BMI z-score and OWO risk (age range: 2-15 years, n=1271).

Maternal BMI	Child Lead (μg/dl)	n	Child BMI z-score mean±SD	β	se	p	Child OWO Case, n (%)	OR	95%CI	P
	<2	679	0.74±1.23	ref			295(43.5)	1.00		
	2-4	526	0.72±1.18	-0.01	0.07	0.831	214(40.7)	0.88	0.69-1.12	0.303
	≥5	66	0.81±1.21	0.15	0.15	0.314	31(47.0)	1.26	0.74-2.15	0.397
	P trend					0.504				0.937
**Stratified**										
Non-OWO	<2	312	0.44±1.22	ref			105(33.7)	1.00		
	2-4	262	0.39±1.19	-0.05	0.10	0.613	74(28.2)	0.74	0.51-1.07	0.107
	≥5	26	0.44±1.12	0.17	0.25	0.504	10(38.5)	1.43	0.60-3.45	0.421
OWO	<2	367	1.00±1.18	ref			190(51.8)	1.00		
	2-4	264	1.05±1.07	0.05	0.10	0.599	140(53.0)	1.04	0.74-1.46	0.810
	≥5	40	1.05±1.22	0.11	0.19	0.554	21(52.5)	1.12	0.56-2.22	0.754
**Combined**										
Non-OWO	<2	312	0.44±1.22	ref			105(33.7)	1.00		
	2-4	262	0.39±1.19	-0.03	0.10	0.735	74(28.2)	0.77	0.53-1.11	0.157
	≥5	26	0.44±1.12	0.16	0.24	0.504	10(38.5)	1.49	0.64-3.50	0.357
OWO	<2	367	1.00±1.18	0.48	0.09	<0.001	190(51.8)	1.93	1.40-2.67	<0.001
	2-4	264	1.05±1.07	0.51	0.10	<0.001	140(53.0)	1.96	1.38-2.79	<0.001
	≥5	40	1.05±1.22	0.56	0.19	0.004	21(52.5)	1.99	1.00-3.95	0.050

Abbreviation: BMI, body mass index; OWO, overweight or obesity.
Analyses were performed by linear and logistic regression models for outcome BMI z-scores and OWO, respectively. Adjusted for maternal education, parity, smoking, race, diabetes, hypertensive disorders, preterm, fetal growth, breastfeeding, and maternal lead in red blood cells.

eTable 11. The individual and combined associations of maternal RBC lead and child blood lead levels on child BMI z-score and overweight or obesity (OWO) risk (age range: 2-15 years, n=1271).

Maternal	Child		Child BMI z-score				Child OWO			
**Lead (µg/dl)**	**Lead (µg/dl)**	n	mean±SD	B	se	p	Case, n (%)	OR	95%CI	P
	<2	679	0.74±1.23	Ref			295(43.5)	1.00		
	≥2	592	0.73±1.18	0.05	0.07	0.483	245(41.4)	0.98	0.77-1.24	0.869
<2		440	0.59±1.26	Ref			165(37.5)	1.00		
≥2		831	0.82±1.17	0.20	0.07	0.004	375(45.1)	1.37	1.06-1.76	0.017
**Stratified by maternal Pb**										
<2	<2	281	0.65±1.30	Ref			111(39.5)	1.00		
	≥2	159	0.47±1.18	-0.00	0.12	0.969	54(34.0)	1.00	0.64-1.56	0.990
≥2	<2	398	0.81±1.17	Ref			184(46.2)	1.00		
	≥2	433	0.83±1.17	0.05	0.08	0.502	191(44.1)	0.94	0.70-1.25	0.655
**Stratified by child Pb**										
<2	<2	281	0.65±1.30	Ref			111(39.5)	1.00		
≥2		398	0.81±1.17	0.13	0.09	0.180	184(46.2)	1.34	0.95-1.88	0.091
<2	≥2	159	0.47±1.18	Ref			54(34.0)	1.00		
≥2		433	0.83±1.17	0.30	0.11	0.004	191(44.1)	1.48	0.98-2.23	0.064
**Combined**										
<2	<2	281	0.65±1.30	ref			111(39.5)	1.00		
	≥2	159	0.47±1.18	-0.05	0.11	0.673	54(34.0)	0.93	0.61-1.42	0.721
≥2	<2	398	0.81±1.17	0.15	0.09	0.093	184(46.2)	1.36	0.98-1.90	0.065
	≥2	433	0.83±1.17	0.21	0.09	0.022	191(44.1)	1.29	0.93-1.80	0.126

Abbreviation: BMI, body mass index; OWO, overweight or obesity.
Analyses were performed by linear and logistic regression models for outcome BMI z-scores and OWO, respectively.
Adjusted for maternal education, parity, smoking, race, pre-pregnancy overweight or obesity, diabetes, hypertensive disorders, preterm, fetal growth, breastfeeding.

eTable 12. The individual and combined associations of maternal RBC lead and child blood lead levels on child leptin and insulin z-scores.

**Maternal Lead (μg/dl)**	**Child Lead (μg/dl)**	n	**Ln leptin** mean±SD	β	se	p	n	**Ln insulin** mean±SD	β	se	p
	<2	378	0.67±0.99	ref			238	2.48±0.78	ref		
	2-4	338	0.59±1.04	-0.03	0.08	0.687	264	2.42±0.87	-0.05	0.07	0.501
	≥5	44	0.66±1.12	0.11	0.16	0.505	28	2.40±0.70	-0.02	0.16	0.896
<2		252	0.48±0.98	ref			164	2.36±0.80	ref		
2-4		424	0.62±1.02	0.15	0.08	0.071	299	2.44±0.79	0.08	0.08	0.315
≥5		146	0.83±1.06	0.37	0.11	<0.001	109	2.65±0.87	0.28	0.10	0.007
**Combined**											
<2	<2	136	0.58±0.93	ref			81	2.39±0.84	ref		
	2-4	84	0.43±1.02	-0.13	0.14	0.348	62	2.29±0.76	-0.14	0.13	0.300
	≥5	10	0.23±1.28	-0.38	0.33	0.243	7	2.12±0.69	-0.41	0.31	0.192
2-4	<2	194	0.69±1.00	0.10	0.11	0.358	125	2.49±0.72	0.03	0.11	0.804
	2-4	177	0.58±1.06	0.05	0.11	0.689	139	2.39±0.88	-0.03	0.11	0.768
	≥5	21	0.52±0.77	0.10	0.24	0.667	13	2.45±0.74	0.19	0.24	0.420
≥5	<2	48	0.81±1.12	0.22	0.17	0.197	32	2.69±0.83	0.19	0.17	0.252
	2-4	77	0.79±1.00	0.26	0.15	0.071	63	2.62±0.94	0.19	0.14	0.163
	≥5	13	1.21±1.32	0.80	0.29	0.007	8	2.56±0.66	0.15	0.30	0.607

Analyses were performed by linear regression models.
Adjusted for maternal education, parity, smoking, race, pre-pregnancy overweight or obesity, diabetes, hypertensive disorders, preterm, fetal growth, breastfeeding, child's age and sex, and maternal lead in red blood cells.
Leptin and insulin were log-transformed.

eTable 13. Maternal plasma folate levels according to prenatal vitamin intake during 3 trimesters.

**Trimester**	**Plasma folate (nmol/L)**			
	≤2 times/wk	3-5 times/wk	Almost daily	P value
First	30.2(20.1-45.3)	33.2(23.5-45.2)	32.5(22.5-44.4)	0.322
Second	25.3(16.4-43.3)	33.7(24.0-46.0)	32.5(23.2-44.5)	<0.001
Third	25.4(16.2-46.6)	34.0(24.7-45.6)	32.6(23.3-45.2)	<0.001

eTable 14. Role of maternal folic acid intake during 2nd trimester in the associations of maternal RBC-lead levels and child BMI z-score and overweight or obesity (OWO) risk (age range: 2-15 years), stratified by maternal OWO status.

**Maternal status**		**Offspring BMI z-score**					**Offspring OWO**			
**Lead (μg/dl)**	**Folic acid Intake (times/wk)**	n	mean±SD	β	se	p	Case, n(%)	OR	95%CI	p
				**Non-OWO mothers (n=636)**						
<2	≤ 2	24	0.33±1.10	ref			5(20.8)	1.00		
<2	≥3	220	0.16±1.16	0.02	0.24	0.949	53(24.1)	1.59	0.51-4.94	0.424
≥2	≤ 2	28	0.21±1.52	ref			7(25.0)	1.00		
≥2	≥3	364	0.57±1.16	0.38	0.23	0.097	132(36.3)	1.74	0.71-4.29	0.227
				**OWO mothers (n=699)**						
<2	≤ 2	28	1.08±1.04	ref			16(57.1)	1.00		
<2	≥3	209	0.93±1.24	-0.21	0.23	0.368	101(48.3)	0.71	0.30-1.70	0.445
≥2	≤ 2	33	1.46±0.96	ref			24(72.7)	1.00		
≥2	≥3	429	1.01±1.12	-0.50	0.20	0.011	224(52.2)	0.38	0.17-0.84	0.017

Abbreviation: BMI, body mass index; OWO, overweight or obesity.
Analyses were performed by linear and logistic regression models for outcome BMI z-scores and OWO, respectively.
Analyses were restricted in folic acid intake during 2nd trimester available samples (n=1335). BMI, body mass index; Adjusted for maternal education, parity, smoking, race, diabetes, hypertensive disorders, preterm, fetal growth, breastfeeding, and child blood lead.

eTable 15. Role of maternal folic acid intake during 3rd trimester in the associations of maternal RBC-lead levels and child BMI z-score and overweight or obesity (OWO) risk (age range: 2-15 years), stratified by maternal OWO status.

**Maternal status**			**Offspring BMI z-score**				**Offspring OWO**			
**Lead (μg/dl)**	**Folic acid Intake (times/wk)**	n	mean±SD	β	se	p	Case, n(%)	OR	95%CI	p
				**Non-OWO mothers (n=634)**						
<2	≤ 2	34	0.29±1.13	ref			8(23.5)	1.00		
<2	≥3	209	0.16±1.17	-0.01	0.21	0.949	50(23.9)	1.21	0.48-3.03	0.684
≥2	≤ 2	26	0.52±1.51	ref			9(34.6)	1.00		
≥2	≥3	365	0.54±1.17	0.03	0.24	0.891	129(35.3)	1.00	0.42-2.35	0.995
				**OWO mothers (n=692)**						
<2	≤ 2	37	1.02±1.10	ref			21(56.8)	1.00		
<2	≥3	196	0.92±1.23	-0.22	0.21	0.300	94(48.0)	0.63	0.29-1.38	0.251
≥2	≤ 2	41	1.28±1.05	ref			25(61.0)	1.00		
≥2	≥3	418	1.02±1.12	-0.34	0.18	0.055	22(52.9)	0.63	0.32-1.23	0.175

Abbreviation: BMI, body mass index; OWO, overweight or obesity.
Analyses were performed by linear and logistic regression models for outcome BMI z-scores and OWO, respectively.
Analyses were restricted in folic acid intake during 3rd trimester available samples (n=1326). BMI, body mass index; Adjusted for maternal education, parity, smoking, race, diabetes, hypertensive disorders, preterm, fetal growth, breastfeeding, and child blood lead.
